# The Current Advancement in Psoriasis

**DOI:** 10.7759/cureus.47006

**Published:** 2023-10-13

**Authors:** Aishwarya P Bhagwat, Bhushan Madke

**Affiliations:** 1 Dermatology, Jawaharlal Nehru Medical College, Datta Meghe Institute of Higher Education and Research, Wardha, IND

**Keywords:** factors, irritation, prevalence, itching, scales, skin inflammation

## Abstract

The study on psoriasis disease helps improve the condition and treatment options day by day in dermatology. In the current scenario, research is ongoing to make the best interventions possible for managing the disease. Psoriasis is one of the most common dermatological conditions wherein chronic inflammation of the skin occurs, characterized by the formation of a rash with scaly, itchy patches over the body surface. The condition is mainly related to the immune system wherein epidermal hyperplasia occurs with infiltration of immune cells. Many factors can trigger psoriasis. Environmental, emotional, hereditary, and personal habits are responsible for the conditions. The current research helps to gain a complete understanding, and the basic knowledge of the state with recent advancements in treatment strategies and characteristic features can also be studied. The main aim is to know the disease's primary root cause and management. It involves the main sites of the body. The most commonly affected parts are the scalp, trunks, knees, elbow, and ankle. It is a chronic long-term disease. Sometimes it may be painful. Itching, bleeding, and disturbed sleep patterns are common symptoms. Most commonly involves the upper epidermal surface. The prevalence of the condition has been increased as it is associated with other comorbidities. The main focus of the treatment is to restrict skin cell multiplication and remove scaly surfaces. This review discusses the advancement in the treatment, its initiation, progression, current symptoms, and disease development. The study also includes basic knowledge about the types of psoriatic disease with its specific features and mechanisms.

## Introduction and background

A chronic inflammatory skin condition known as psoriasis has autoimmune pathogenic characteristics and a high immune-mediated genetic susceptibility [1]. Acquired and cellular immunity interact intricately in pathophysiological pathways [2]. The disease has complex multifactorial etiology affecting the population. Most frequently, the condition is brought on by hereditary and external factors. The prevalence of the disorder correlates with its severity. Psoriasis vulgaris is a term used for psoriasis. However, it is also referred to as plaque-type psoriasis. The patchy appearance is seen all over the extremities. The chronic plaque type of psoriasis is present in about 90% of patients. Classical clinical signs include patches with distinct borders that are erythematous, pruritic, and encased with silvery scaling [1].

Large patches of skin may become covered by the plaques when they consolidate. The typical sites involved are upper limb, lower limb, scalp, trunks, etc. They include psoriasis vulgaris, psoriasis of inverse type, blister psoriasis, and so on. It is common in all age groups, from children to adolescents. The PSORS1-9 locus is one of many inherited risk loci discovered in genome-wide association studies [3]. Psoriasis has been demonstrated to have a broad spectrum of comorbidities in adults, primarily biochemical and cardiac conditions, as well as anxiety, stress, depression, and autoimmune diseases [3]. Psoriatic arthritis (PsA), metabolic disease, heart issues, endocrinopathies, and other comorbidities are frequently linked to the condition. Patients also have chronic renal problems and chronic inflammatory bowel disease. Additionally, there are more cases of anxiety and suicidality. Streptococcal infections, physical trauma (e.g., tattoos and surgical incisions), specific medications (e.g., antidepressants, antihypertensive meds, anti-cytokine therapy), cigarettes, and alcohol addiction are a few examples of known external factors and correlations [4].

## Review

Psoriasis and genetics

**Figure 1:**
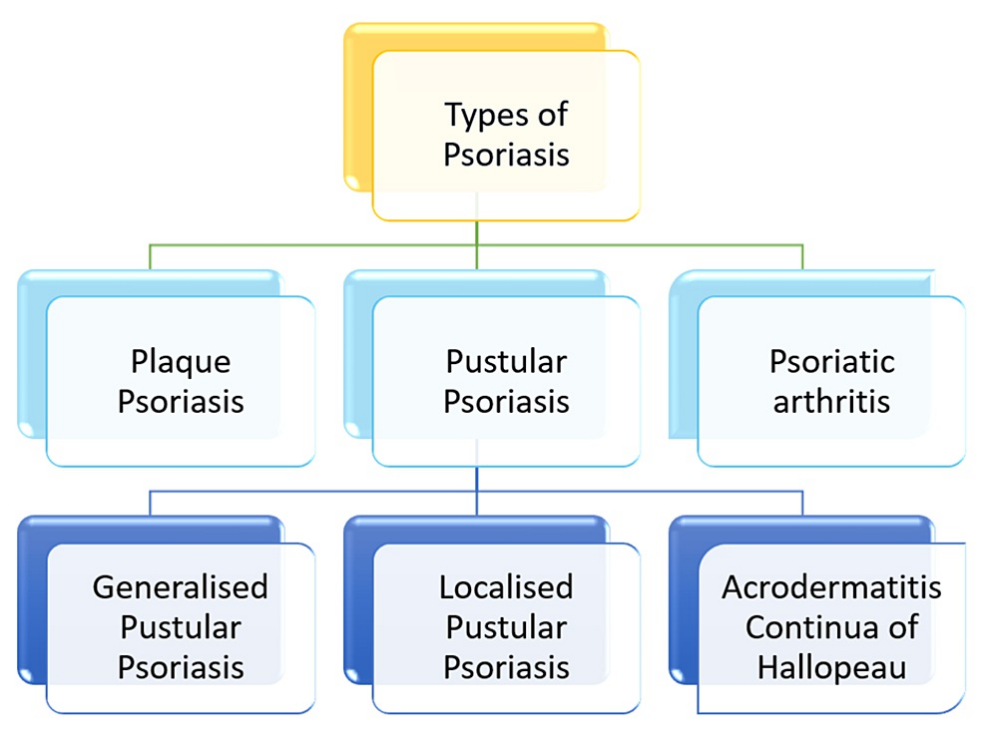
Various forms of psoriasis. The figure was created by the authors themselves.

Psoriasis is common in all age groups. The genetic factor thus plays an important role. Recent discoveries about the molecular basis of the widespread skin condition psoriasis highlight the opportunity for genetic studies to be translated into new and effective biological treatments and small chemical inhibitors [5]. Psoriasis has an exceptionally high hereditary element among complicated conditions, with inheritance reported to be above 60%. It is more common in monozygotic twins than in dizygotic ones. The prevalence is higher in first- and second-degree relatives with psoriasis than in general. In polymorphic pedigrees, research on linkage discovered at least nine genomic regions (loci) called psoriasis susceptibility 1 locus (PSORS1-9) that correlated with psoriasis. The highly improved microarrays used in genome-wide association studies (GWAS) can reliably and effectively genotype millions of genetic loci throughout the human genome. Compared to linkage analysis, GWAS is substantially more effective as it can identify even minute changes in the frequency of alleles between disease patients and unaffected subjects. Extensive serological tests were mainly used to determine zygosity. The analysis results demonstrate that the particular genotype's existence is almost entirely necessary for the beginning of the disease [6]. On chromosome 17q, the PSORS2 gene is close to the telomeric end. In several investigations, the risk allele's precise location has varied somewhat [7]. The central histocompatibility complex region at 6p21 has significantly the highest hereditary threat of psoriasis. The type I human leukocyte antigens (HLA) gene HLA-C exhibits a consequential reliance of vulnerability to psoriasis throughout the major histocompatibility complex (MHC) [8]. The transformative utilization of GWAS data is receiving a lot of interest because of the efficacy of GWAS analysis. Over 10,000 loci have been found so far through GWASs. However, genetic variation (i.e., heritability) by such extensive GWAS data does not match that discovered in twin research. For instance, the newest P-value in genome-wide association studies (PV GWAS) results account for around 30% of total inheritance. Therefore, more advancements in the sequencing process are required before large-scale GWASs can be regularly conducted [5]. Figure 1 explains the type of psoriasis mentioned in the given review.

Plaque psoriasis

Pathogenesis

Plaque psoriasis is a cutaneous condition that causes dry, itchy, red erythematous, raised skin patches owing to the progression and differentiation of keratinocytes. The complete mechanism of psoriasis occurs in the initiation and maintenance phases. In the initiation phase, the principal adenosine mono phosphate at play is LL37, which initiates the process by interacting with either deoxyribonucleic acid or ribonucleic acid (DNA or RNA). It triggers a toll-like receptor (TLR) when interacting with DNA; TLR stimulates proliferative dendritic cells (pDCs). Activated pDCs produce IFN-α and IFN-ß. Interferons produced by pDCs thus recognized myeloid dendritic cells (mDCs) to expand further and differentiate type 1 and 17 helper cells. T1h cell produces tumor necrosis factor alfa (TNF-α), and T17h cell produces IL-17 and IL-22. The main pro-inflammatory cytokine is TNF-α, which is produced by Th1 and Th17 cells. When the AMP, LL37, binds to RNA, it stimulates TLR7 and TLR8. It also secretes TNF-α, IL-23, and ß-design, which enhances the RNA and DNA recognition. During this binding, neutrophils are increased in plaque psoriasis. Helper T cell subgroups and their associated cytokines play a significant role in the maintenance phase. IL-23/IL-17 plays a substantial part in the pathophysiology of psoriasis. Psoriatic lesions contain large amounts of IL-23, which keeps Th17 cells alive. The innate immune system may produce IL-17 without the aid of IL-23. Contrary to typical T cells and gamma and delta chains, T cells particularly have a TCR composed of alfa and beta chains, and they are more prevalent in psoriatic skin lesions. According to a TCR screening, gamma and delta T cells comprise a fraction of one percent of all T cells found in psoriatic lesions. The number of ILC3s that are NCR+ positive (natural cytotoxicity receptor positive) is also shown to be higher in psoriatic lesions. Interleukin C, natural cytotoxicity receptor+positive ILC NCR+ is stimulated in vitro, producing IL-22, which has been linked to the pathophysiology of psoriasis. Skin with psoriasis contains natural killer T cells (NKT) cells, which disappear once treated. Additionally, transplanting NKT cells causes the development of psoriasis, indicating that they may play a part in the disease's pathophysiology [9].

Clinical Features

It appears as reddened spots or patches with distinct borders. Due to enhanced blood circulation in the outermost layer, an overlying silvery-white scale may be seen, and removal may result in pinpoint bleeding. White streaks represent leukonychia. The nails are frequently affected by psoriasis, with an average prevalence of 80-90%. Nail psoriasis is related to a more severe illness that manifests more quickly and has a higher risk of developing PsA. Pink spots, subungual hyperkeratosis, onycholysis, and splinter hemorrhages are specific symptoms of nail psoriasis. Under the nail plate, pink patches appear as erratic, discolored, yellow-orange regions. Linear reddish-brown spots of principal bleeding in the nail matrix are splinter hemorrhages. It also affects the extremities' extensor regions, such as the scalp, elbows, knees, eyelashes, and gluteal folds. Inverted psoriasis (i.e., longitudinal psoriasis) is also plaque-like psoriasis [10].

Pustular psoriasis

This is a therapeutically diverse group of distinct disease subgroups, including generalized pustular psoriasis and palmoplantar psoriasis, which are the most familiar. These variants may be linked to plaque psoriasis symptoms despite being phenotypically and genetically isolated from psoriasis vulgaris, providing support for being included in the dermatitis spectrum. It is present most commonly in the form of aseptic pustules. They are produced because of the mutation of any of the three genes, mainly interleukin 36 receptor antagonist (IL36RN), caspases recruitment domain (CARD14), and adaptor-related protein complex 1 subunit sigma 3 (AP1S3). With interleukin-36 agonists serving as the most progressed example of therapeutic research, these recent developments have sparked the planning of biological medicines that directly target the leading causes of inflammation in pustular psoriasis. Clinical signs and symptoms are defined based on generalized pustular psoriasis, localized pustular psoriasis, and acrodermatitis pustular psoriasis [11].

Generalized pustular psoriasis (GPP)

Asian origin has a higher incidence rate of generalized pustular psoriasis. It can affect anyone at any age and is more prevalent in women than in males, but it repeatedly targets people in their fourth and fifth phases of life [12]. It has a large skin rash characterized by aseptic abscesses, and a varied degree of fever is the acute beginning symptom, which is later joined by general malaise, exhaustion, and other systemic manifestations. There may be additional signs, such as polyarthralgia, polymyalgia, and rheumatism. It mainly involved areas with large skin folds. The skin is sore and sensitive. It takes only a few hours for tens to hundreds of sterilized non-follicular pustules to emerge. As they grow, they may congregate to form pus pools. The GPP is caused by hereditary factors, selected gene loci, primarily inflammatory cytokine signaling elements, and external factors (e.g., virus infections, medicines, and stopping corticosteroids) [13]. GPP is typically categorized as a psoriasis variation. However, the former's differential therapeutic, histological, and inherited features imply that the pathogenic mechanisms underlying each of these illnesses are, at least in part, dissimilar [14]. Systemic medicines that are biological and nonbiologically based can currently be used to treat GPP nonbiological systemic therapies that include drugs like methotrexate, cyclosporine, and retinoids. Moreover, biological treatment includes infliximab and adalimumab [15].

Localized pustular psoriasis

It is also called palmoplantar pustulosis (PPP). People with pustular psoriasis experience tiny skin blisters filled with pus. Affected body parts may include extremities in cases of pustular psoriasis. It is referred to as palmoplantar pustulosis [16]. The most widespread type of pustular psoriasis, known as PPP, has a prevalence of between 0.05 and 0.12% and is noticeably more common in Japan. It is more common in women between 50 and 69 years. A chronic irritating illness called palmoplantar pustulosis (PPP) causes patches of benign blisters with reddened keratotic plaques that bleed and cause pain in the palms and soles. PPP is a reactive, long-standing condition that causes severe pain and pruritus, significantly reducing the standard of living in terms of health. Patients may report excruciating scorching pain. There are frequently brown macules and scaling when the sterile pustules heal. The nails could be impacted as well. Synovitis, acne, pustulosis, hyperostosis, and osteitis (SAPHO) syndrome are conditions linked to PPP obesity, and diabetes, dyslipidemia, problems with the thyroid, and other comorbid diseases have also been linked to PPP. However, more extensive studies are required to validate these associations. PPP may develop because of external aggravating factors like smoking, drug abuse, stress, localized diseases, and mental allergies. In PPP, genetic predisposition is significant [17]. Various medications (e.g., adalimumab, infliximab, ustekinumab, methotrexate, cyclosporine, and etretinate), light therapy, and topical vitamin D topical corticosteroid were the different categories for treating PPP [18].

Acrodermatitis continua of hallopeau (ACH)

It is an uncommon type of psoriasis that affects the phalanges of hands and feet; successive crops of blisters merge on the nail bed and matrix, forming streams of pus, leading to dystrophy of the nails along with anonychia of the affected digits. A chronic condition with periodic recurrence of ACH can have hazardous and devastating effects, especially when it coexists with PsA [19].

PsA

PsA affects 20-30% of people. PsA is an inflammatory muscular condition linked to psoriasis of the skin. Men and women are affected similarly between the ages of 40 and 50. The various systemic organs impacted include the epidermis nails, axial and lateral joints, and bones. PsA is related to systemic conditions such as bone deformity, uveitis, intestinal swelling, and cardiac abnormalities [20]. Poorer physical health has been linked to delayed PsA diagnosis. The potential of "personalized medicine" could encourage additional advancements in treatment quality in the future [21]. Clinical features are as follows, increasing the risk of PsA depending on the severity of psoriasis. The risk of PsA with the gluteal and the perianal region is three times higher, and the risk of that of the scalp region is threefold. When determined by a clinical examination, enthesitis is one of the primary characteristics of PsA, with a prevalence of about 30%. The term "enthesitis" denotes the presence of inflammation at the enthesis [22]. In treatment over the past several years, the number of pharmacological therapies for PsA has increased significantly; however, long-range therapeutic outcomes are generally determined by clinical trials rather than thorough head-to-head trial-based evaluation. Targeted biological therapies have replaced conventional disease-modifying antirheumatic drugs (DMARDs) as the primary symptomatic therapy throughout the past 20 years. About 50% of patients have normal c-reactive protein and erythrocyte sedimentation rates [23].

Treatment of psoriasis

Past Treatment

Psoriasis was understood to be a recurring and transferring skin disorder that might be temporarily cured with treatment but not entirely cured. It is widely believed that treating psoriasis as a standalone skin condition is no longer appropriate. While choosing a course of treatment, other options should be selected based on the severity of the involvement of the dermis. The existence of PsA and risk factors, clinical and social effects, concurrent drugs, planned pregnancy, personal preferences, and treatment objectives are among them. An illustration of successful transferable research is the growth of increasingly accurate targeting of medicines for psoriasis over the last century. This has been made possible by improved knowledge of the biology of the illness. Even 100 years ago, there were numerous systemic and topical psoriasis treatments accessible, including the use of salicylic acid, tar from coal, and dithranol preparations. Topical steroids are the first to be reported as used in patients with psoriasis. Biologics are the proof of concept in this contemporary drug development method and patient treatment. Due to its low potency, there was no discernible effect, which could have been the case. Orally administered prednisolone and triamcinolone are effective. In the 1980s, vitamin D analogs were researched for various dermatoses. When applied topically to treat psoriasis, calcipotriol was demonstrated to help reduce the progression of epidermal keratinocytes. It became clear that psoriasis needed a reliable and unbiased method for assessing the severity and response to treatment as more systemic treatments were developed [24].

Present and Future Management

Biologics are recommended in moderate to severe psoriasis where systemic therapy fails. This licensing supports the present stepwise method of treating psoriasis. Initially, topical treatment is often administered to patients with mild or limited disease. If this is insufficient, they are diagnosed with a moderate-severe illness and may benefit from phototherapy or other traditional systemic treatments. Currently, available biologic therapy includes certain drugs such as TNF-α inhibitors (Infliximab, adalimumab, certolizumab pegol, etanercept), IL23 inhibitors (ustekinumab, guselkumab, risankizumab, and tildrakizumab), IL17 inhibitor (secukinumab, ixekinumab, brodalumab), IL36 receptor antagonist, sphingosine one phosphate (S1P) agonist (ponesimod), rho-associated kinase inhibitor (ROCK2), the AHR agonist (topinarof), and Janus kinase inhibitor (JAK) (tofacitinib).

TNF-α inhibitor: It is acknowledged as a crucial regulatory cytokine in psoriasis and other chronic immune-related inflammatory disorders. The first TNF inhibitor authorized for the treatment of psoriasis was etanercept. TNF inhibitors, infliximab, adalimumab, and certolizumab pegol are currently offered for treating psoriasis. TNF has a significant function in the induction and transmission of immunological activity by focusing on an inflammatory response that penetrates the epidermis, and it is present in high amounts in lesions on the skin and plasma of psoriasis patients [25]. Adalimumab is now licensed by the Food and Drug Association (FDA) and European Medicines Agency (EMEA) for the management of PsA, for which it is given via a subcutaneous route every week. It is an immunoglobulin G1 monoclonal antibody and recombinant; it binds to TNF with great sensitivity and affinities [26].

Among the IL23 inhibitors, ustekinumab is the first inflammatory drug approved for psoriasis. It attacks the p40 subunit of the interleukin 23. In the short-term management of moderate to severe psoriasis, ustekinumab is much more efficacious than placebo and etanercept [27]. Guselkumab is a monoclonal antibody (mAb) made entirely of human immunoglobulin G1 (IgG1) that binds to the p19 component of IL-23. Guselkumab has also been or is now being explored for the treatment of other disorders, including generalized pustular psoriasis (GPP), erythrodermic psoriasis (EP), and PsA, in light of its clinical effectiveness in plaque psoriasis [28]. Risankizumab has greater efficacy in treating the condition compared to adalimumab as it has been proven by a meta-analysis of the case [29]. Tildrakizumab must be injected subcutaneously. The medication is absorbed gradually. Steady-state quantities are attained at 16 weeks in the suggested schedule of one injection next to another after four weeks, following that every 12 weeks [30]. Regarding IL17 inhibitors including secukinumab, ixekizumab, it is now known that other cell types, including T lymphocytes and innate lymphoid cells (ILC) 3, are also significant sources of IL17 in the skin in response to inflammatory stimuli. IL17 specific has been called a T helper (Th) 17-produced cytokine [31]. One of the multiple targeted biologic treatments newly licensed for treating plaque psoriasis in pediatric patients is subcutaneous secukinumab. Secukinumab binds to IL-17A and prevents its production of cytokines and chemokines that promote inflammation. Children and adults with moderate to severe plaque psoriasis, aged 6 to 18 years, responded quickly and permanently to treatment with secukinumab, which also led to ongoing improvements in health-related quality of life [32]. Ixekizumab has shown continued effectiveness in the treatment of psoriasis of the skin and nails. Based on comparison studies with comparative therapies, ixekizumab has shown great significance in treating plaque psoriasis [33]. In the long-term open-label phases of the psoriasis program, brodalumab was administered to every participant who committed suicide [34]. The primary side effects of brodalumab were candidiasis, neutropenia, inflammatory bowel disease, depression, and an increased suicide risk.

The inflammatory mediator agonists of IL36 are IL-36 and IL-36R, as well as another receptor antagonist (IL-36Ra), and they are members of the IL-36 group of cytokines. These cytokines adhere to and transmit through a heterodimeric transmitter of IL-36R and the IL-1R accessory protein (IL-1RAcP) [35]. One of the IL36 receptor antagonists is spesolimab for the treatment of generalized pustular psoriasis (GPP) flares in adults, and spesolimab has received approval in the USA following the first dose of spesolimab. A second dose of 900 mg was given for one week if GPP flare symptoms were still present. The recommended dosage of spesolimab is 900 mg as an intravenous infusion over 90 minutes [36]. Sphingosine 1 phosphate (S1P) agonist (ponesimod) is a multifunctional bioactive lipid known as sphingosine-1-phosphate (S1P), and it is involved in a variety of physiological and pathological processes. S1P is a signaling molecule communicating with S1P receptors, and it is essential for controlling immunological conditions such as psoriasis. Ponesimod, siponimod, and ozanimod are directly acting sphingosine modulators. Ponesimod improves a majority of sufferers by lowering the degree of involvement and areas affected. Moreover, it contributes to the persistence of lymphocytes in damaged tissue [37].

Rho-associated kinase inhibitor (ROCK2): The phosphorylation of downstream targets in cells is mediated by the rho family kinases ROCK1 and ROCK2, which are serine-threonine kinases [38]. Depending on the person's illness status, the role of ROCK proteins in controlling the immune system may vary [39].

The aryl hydrocarbon receptors (AHR) agonist (tapinarof): For the treatment of psoriasis and atopic dermatitis, tapinarof, a new, first-in-class, small-molecule topical therapy aryl hydrocarbon receptor (AhR)-modulating drug, is currently undergoing clinical testing. The particular linking and stimulation of AhR a is credited with tapinarof's ability to treat psoriasis [40]. Tapinarof activates Ahr, which controls T helper cell development, which inhibits IL-17 production and lessens inflammation in psoriatic skin [41].

Janus kinase inhibitor (JAK) (tofacitinib): tofacitinib, an effective psoriasis treatment, has recently been combined with one of the first regulators of signaling proteins directly connected to cytokine receptors [42].

Biologics plays an important role in treating this condition [25-42]. Table 1 shows important biologics.

**Table 1:** Various biologics used in the treatment of psoriasis. The table was created by the authors themselves.

Biologics	
TNF-α inhibitor	Infliximab, adalimumab, and certolizumab pegol, etanercept
IL23 inhibitors	Ustekinumab, guselkumab, risankizumab, and tildrakizumab
IL17 inhibitor	Secukinumab, ixekinumab, brodalumab
IL36 receptor antagonist	Ponesimod, sepsolimab, siponimod
AHR agonist	Topinarof
Janus kinase inhibitor (JAK)	Tofacitinib

Physical complications that affect life expectancy in psoriasis

Psoriasis is a condition that is also related to other comorbidities such as obesity, dyslipidemia, cardiovascular diseases, PsA, and so on. Psoriasis has since been proven to be a systemic and autoimmune-sensitive illness that may be linked to additional diseases of an inflamed nature. New studies have shown the function of cytokines and mediators in the genesis of disease. Dyslipidemia and obesity in people with psoriasis can lower lifespans and increase the chances of systemic and metabolic conditions; hence, it is imperative to routinely investigate such complications in psoriasis patients. Recent research has shown that obesity has a mildly pro-inflammatory state with elevated amounts of tumor necrosis factor and IL-6, which may cause or aggravate lesions related to psoriasis [43]. Severe abnormalities in lipids are present in individuals with psoriasis. Particularly, patients' lower-density lipoprotein and higher-density lipoprotein fraction triglyceride contents were significantly greater [44]. Persons with psoriasis have higher incidence rates of cardiovascular diseases, such as cigarette use, preclinical coronary artery disease, diabetes mellitus, obesity, high blood pressure, and lipid abnormalities. If a patient needed systemic medication such as methotrexate and biological agents, they were considered to have serious psoriasis. There is a strong correlation between the prevalence of heart disease and the financial and medical burden [45].

Physical complications due to changes in the immune system caused by therapeutic drugs in the treatment of psoriasis

Specific biologics are used to treat comorbidities and other systemic diseases, but some of the therapeutic drugs can cause complications. The treatment plans should be customized to match unique needs depending on the severity of the condition, how it affects the quality of life, how well it responded to previous medications, and whether there are any coexisting conditions [46]. In some vascular diseases, psoriatic treatment leads to thrombosis. Biological drugs can also lead to renal complications, unresponsiveness, irritation, and depression as they affect the quality of life. The current generation of biological pharmaceuticals is traditionally thought to be harmless in patients with previous malignancy, even if only research studies and brief reports on the administration of biological medicines in the public are found to be unresponsive as they occasionally cause serious malignancy [47].

The impact of psoriasis on cardiovascular diseases, metabolic syndrome, and stroke

Cardiovascular Diseases

Traditionally thought of as a dermatological condition, psoriasis is becoming more well-recognized for its systemic effects, particularly its complex relationship with cardiovascular diseases (CVD). There may be a common inflammatory route between CVD and the chronic, immune-mediated inflammation that characterizes psoriasis. Psoriasis sufferers have a higher-than-average risk of cardiovascular events, according to research. Even after controlling for conventional cardiovascular risk factors, crucial research by Gelfand et al. found that individuals with severe psoriasis had a 1.5- to 2-fold higher risk of myocardial infarction [48]. This important study emphasized the systemic nature of psoriasis and the demand for a deeper comprehension of its effects outside of the skin. Studies have also probed the possible pathways between psoriasis and CVD. Psoriasis' chronic inflammation may be a factor in endothelial dysfunction, insulin resistance, and dyslipidemia, all of which are essential for the growth of atherosclerosis and subsequent cardiovascular events. The Nurses' Health Study II results highlighted the systemic influence of this skin condition on metabolic health by showing a dose-response association between the severity of psoriasis and the likelihood of acquiring type 2 diabetes [49]. In addition to conventional risk factors, the length and severity of the psoriasis condition have been associated with an elevated risk of CVD. A more prominent systemic inflammatory load may be present in patients with severe psoriasis, especially in those with an early age of start, which adds to the cardiovascular risk profile. This emphasizes the need to include psoriasis as a separate risk factor when assessing cardiovascular risk.

In conclusion, research into the complex connection between psoriasis and cardiovascular disorders is a growing field with important therapeutic ramifications. To give patients holistic treatment, healthcare professionals must have a thorough awareness of this relationship. Optimizing the long-term health outcomes of people with psoriasis requires not just treating cutaneous symptoms but also controlling cardiovascular risk factors.

Stroke

Psoriasis, a chronic inflammatory skin illness, is becoming more widely acknowledged for its potential systemic effects, including a suspected connection to cerebrovascular events, such as strokes, and is increasingly seen as more than just a dermatological problem. Even while more study is needed to fully understand this connection, mounting data points to a complicated interaction between the risk of stroke and the inflammation associated with psoriasis. This relationship is explained by notable research from Taiwan by Tsai et al., which found that people with psoriasis had a higher risk of stroke, especially if they have severe symptoms and concomitant conditions like diabetes and hypertension [50]. The results indicate the possible contribution of psoriasis-related inflammation to the development of both ischemic and hemorrhagic strokes by inducing a prothrombotic state and vascular dysfunction. While the exact causes of the link between psoriasis and stroke are still unknown, it has been hypothesized that the chronic inflammation that is a hallmark of the condition may contribute to endothelial dysfunction and systemic inflammation, both of which are known causes of cerebrovascular events. Furthermore, common risk factors for stroke (e.g., obesity, metabolic syndrome, and hypertension), which are common among psoriasis sufferers, increase the risk of stroke. The possible influence on stroke risk highlights the necessity for a holistic approach to patient care as we work to understand the complex interactions between psoriasis and systemic health. Clinicians should think of psoriasis as an illness with systemic effects rather than only a skin problem, necessitating diligent monitoring and management of cardiovascular risk factors to reduce the risk of stroke in afflicted people. This developing knowledge emphasizes the significance of current studies to identify the precise mechanisms that connect psoriasis and stroke, providing fresh information on potential preventative and therapeutic measures for those who suffer from this chronic skin condition [50].

Metabolic Syndrome

A paradigm change in our understanding of the systemic effects of psoriasis has occurred with the recognition of this disorder's complicated relationship with metabolic syndrome, which has long been known as a chronic inflammatory skin illness. The metabolic syndrome, which is characterized by insulin resistance, dyslipidemia, obesity, and hypertension, is a result of the chronic inflammation found in psoriasis. The revolutionary research of Langan et al. in a thorough investigation conducted in the United Kingdom found a significantly higher frequency of metabolic syndrome in people with psoriasis [51]. The therapeutic importance of identifying and controlling these systemic consequences was also underscored by this investigation, which not only confirmed the link between psoriasis and metabolic abnormalities. The necessity for an integrated approach to patient care is highlighted by the bidirectional association between psoriasis and metabolic syndrome, where the former worsens the latter. Clinicians should focus on lifestyle changes and complete treatment techniques in addition to treating the apparent skin symptoms of psoriasis to improve the general health of those who have it.

## Conclusions

Psoriasis is a well-known dermatological condition; this review contains general information, its types, peculiarities, characteristic features, and management. Psoriasis has a detrimental influence on quality of life, equivalent to other serious illnesses, not just in adults but also in kids and teenagers. According to twin studies, up to 70% of the risk of developing psoriasis can be linked to genetics. Additionally, having one psoriatic parent increases the likelihood that their child would develop the condition by 30%, while both parents raise the incidence by 50%-65%. Pustular psoriasis is a formidable group of autoinflammatory skin conditions with clinical and genetic variability. However, about 100 years after von Zumbusch's ground-breaking description of GPP, the growing cooperation between medical professionals and scientists is optimistic. It enables a better nosological categorization of sub-entities and the creation of specialized therapeutic techniques. Over the past 20 years, PsA research has advanced significantly. New medicinal drugs and treatment modalities have been created, outcomes and assessments have improved, and understanding of physiology has increased. Physicians must be informed about these significant advancements to provide the best results for patients with PsA. Proper measures can help in treating the disease. Current progress in treatment modalities of the disease by many researchers has an excellent impact on knowing the condition.
